# Effect of Undercut Bolt Anchor Depth on Failure Cone Geometry: A Numerical FEM Analysis and Experimental Verification

**DOI:** 10.3390/ma18030686

**Published:** 2025-02-04

**Authors:** Józef Jonak, Andrzej Wójcik, Robert Karpiński, Kamil Jonak

**Affiliations:** 1 Department of Machine Design and Mechatronics, Faculty of Mechanical Engineering, Lublin University of Technology, ul. Nadbystrzycka 36, 20-618 Lublin, Poland; 2 Department of Technical Informatics, Lublin University of Technology, ul. Nadbystrzycka 38D, 20-618 Lublin, Poland

**Keywords:** undercut/breakout anchor, FEM, rock mechanics, rock cone failure, sandstone

## Abstract

This study examined the influence of the effective embedment depth *h*_ef_ of undercut anchors and the diameter of their heads on the formation of the so-called cone failure angle α. Cone failure formation during simulated anchor pull-out tests was analyzed numerically using the Finite Element Method (FEM) with the ABAQUS software and the XFEM algorithm. The analysis was conducted for three sizes of undercut anchor heads and four embedment depths. The numerical analysis results were compared with field test results obtained during pull-out tests of anchors installed in a rock medium (sandstone). Good agreement was observed between the numerical and field test results. The results of the numerical study are highly consistent with those obtained during the field survey. Moreover, they align closely with findings from previous numerical studies conducted by members of the research team, as presented in earlier publications. For the assumed simulation and field test conditions (sedimentary rocks, gray sandstone), no clear correlation was found between the embedment depth or the anchor head diameter and the value of the cone failure angle in the initial phase of the failure zone development. This result contrasts with certain findings reported in the literature. Many existing studies on anchor bolts focus on material properties or load-bearing capacity, but lack an in-depth analysis of how anchor depth influences the geometry of the failure cone. This research addresses that gap, providing valuable insights with practical implications for design codes and safety evaluations.

## 1. Introduction

In the field of infrastructure-fastening technology, such as road infrastructure components in concrete (reinforced concrete) structures, various structural solutions for anchoring elements (anchors) are employed [1,2,3,4]. The most common anchor types include headed bolts, undercut anchors, expansion anchors, and bonded expansion anchors [5,6,7]. The problem of pull-out force generation and the so-called cone failure formation during anchor extraction (including undercut anchors [8,9,10]) has been a subject of interest among researchers for years [11,12,13,14]. This is due to the widespread use of such elements in the installation of diverse technical infrastructure in concrete engineering structures [15,16,17].

The necessity of ensuring the safe operation of these installations requires precise estimation of anchor load capacities (pull-out forces) as well as determining the extent of the failure zone in the medium during potential emergency anchor extraction. The interaction of the rock bolt support with the rock mass under laboratory conditions can be monitored using strain gauges and a self-excited acoustic system [18,19,20]. Studies [21,22] have identified the primary failure modes associated with anchor pull-out under tensile loading, both on the anchor and the medium in which it is installed. These failure modes include the following: steel failure, pull-out failure, concrete breakout failure, splitting failure, and concrete side-face blow-out failure [23,24].

For anchors installed in groups, it is crucial to determine a safe spacing that eliminates interaction effects between anchors, which can reduce the load-bearing capacity of the anchor assembly [25,26]. Research in this area is conducted analytically using linear fracture mechanics and relevant mechanical models [27,28,29,30], numerical methods such as the Finite Element Method (FEM) [31,32,33,34], or the Boundary Element Method (BEM) [35].

In FEM analyses of anchor–medium interaction, the cohesive zone model (CZM) is predominantly employed [36,37]. This model is particularly effective in simulating the behavior of anchors under load, providing insight into the processes leading to material failure and the extent of the failure zone.

Numerous studies have focused on the development of failure zone models (parameters of the failure cone) and the influence of anchoring parameters and the mechanical properties of the medium in which the anchor is installed on the extent of the failure zone during potential anchor pull-out.

In earlier analyses, the failure zone of the medium was modeled as a failure cone, assuming a constant cone angle α = 45° (e.g., Bennett [38]). Based on the developed theory of the failure cone, it was assumed, for instance, in [38,39], that for a certain permissible ultimate stress in concrete (tensile strength) and a constant applied load *F*, the area (lateral surface of the cone) required to resist this force must remain constant.

It was further postulated that if the embedment depth increases while the surface area remains constant, the cone angle α must also increase. Consequently, with an increase in embedment depth, the failure cone angle α is expected to grow.

**Figure 1:**
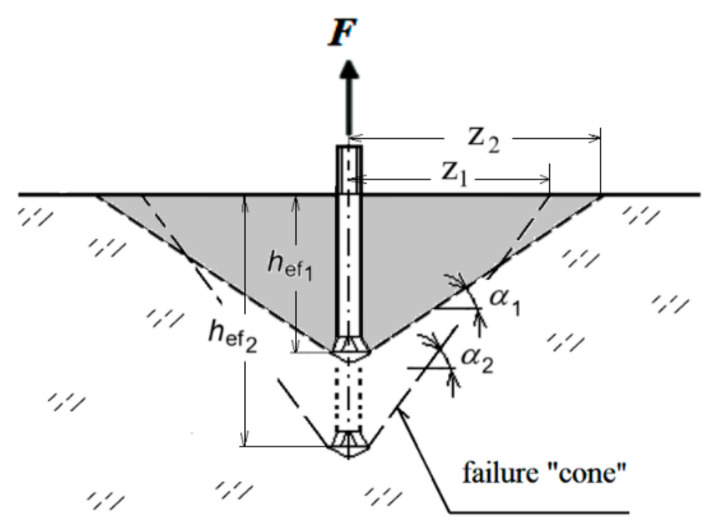
Formation of the geometry (angle) of the destruction cone α depending on the anchoring depth *h*_ef_; *z*_1_, *z*_2_—range of detachment on the free surface for the considered anchoring depths; α_1_, α_2_—angle of the destruction cone corresponding to *h*_ef1_, *h*_ef2_ (with *f*_c_′ = const.).

This problem, in the context of undercut anchors, is illustrated in Figure 1. This representation helps to demonstrate the relationship between embedment depth and the geometry of the failure zone, particularly the dependency of the cone angle α on varying anchoring depths.

According Saleh and Aliabadi [35], increasing the anchoring depth *h*_ef_ from 100 mm to 200 mm resulted in a corresponding increase in the angle α, from approximately 38° to around 44° (the estimation was based on drawings presented in [35]). Jonak et al., in [40], found that the anchoring depth *h*_ef_ significantly influences the pattern of rock medium destruction. It was observed that for small values of this parameter, during the initial phase of penetration, the fracture develops at an angle of α ≈ 21°. As the depth increases, this angle gradually rises, reaching approximately 29° for *h*_ef_ = 200 mm. However, in these studies, which were conducted using the FEM, difficulties arose in unequivocally determining the value of the angle α due to the computational process halting as a result of algorithmic convergence issues.

There are studies indicating that the propagation angle of the destruction surface α depends on the anchoring depth *h*_ef_ (e.g., [41]—Variable Angle Cone (VAC) model). A similar approach to this problem can be found in [42,43]. However, some models assume that the angle α is constant (and, in particular, does not depend on the anchoring depth) [34,44]. At the same time, according to [44], with an increase in ft and a decrease in *h*_ef_, α decreases. The challenge, however, lies in the fact that estimating the destruction cone angle is not a straightforward process [27,43,45].

In later studies, it was assumed that the destruction zone could be described as a cone, but with an angle α = 37.5° (Linear Fracture Mechanics Method—LEFM [27]) or 35° (Concrete Capacity Design Method—CCD [46]). According to the CCD method, this angle is determined at the point when the pull-out force on the anchor reaches its maximum value, corresponding to a point on the trajectory located approximately at a depth of ~0.45 *h*_ef_ [43].

The latest standard recommendations assume that the failure zone can be described as a four-sided pyramid with a failure angle of α = 35°, with the apex at the top of the anchor head and the base at the concrete surface, with a side length of 3 *h*_ef_ (e.g., the CCD method [46,47,48,49]). However, numerous studies demonstrate that the simplifications adopted in the above models lead to significant deviations from the predicted values, both in terms of the estimated pull-out force and the potential extent of the destruction zone (size of the potential failure cone) [30,44,50].

Lu et al., in [37], show that as the embedment depth decreases, the failure cone becomes smaller and steeper. Furthermore, the angle of fracture propagation, this time measured relative to the load direction (90° − α), increases with greater embedment depth. For smaller embedment depths, the cone is steeper compared to larger depths, which is contrary to findings from studies such as [35,40,51].

For a constant embedment depth, the concrete cone is almost independent of the thickness (diameter) of the anchor rod, as demonstrated, for example, in [52] for undercut anchors. According to [53,54], the destruction angle increases with the size of the anchor head diameter. Furthermore, [48] indicates that the head size significantly impacts the tensile capacity of the anchor. A stiffer response from the anchor can be observed with an increase in head size.

Additionally, two distinct primary failure modes of concrete were observed depending on the head size and effective depth. In one of these modes, failure occurred in two phases (Figures 6 and 7 in [48]): initially, a “lower crack I” formed, followed by the formation of “crack II” after reaching the peak reaction, which continued to open until the end of the simulation. Crack I did not progress further after the opening of crack II.

In Yang and Ashour [45], a bilinear propagation shape of the destruction zone was assumed, whereas [44] proposed a hyperboloidal model of the destruction cone associated with the pull-out of “headed bolt” type anchors. However, doubts remain as to whether the hyperboloidal destruction cone model is applicable to other types of anchors, such as undercut anchors. This is linked to the fact that in “headed bolt” anchors, the formation of the destruction cone is assumed to result primarily from tensile stresses.

In existing models, the failure process is predominantly governed by Mode I fractures (tensile fractures), making the cohesive zone model suitable for analyzing the development of the destruction zone (formation of the failure cone) [55]. According to [50], the process of failure cone formation and its geometric parameters depend on the type of loading applied during anchor pull-out (force-controlled or displacement-controlled). Furthermore, ref. [50] states that with an increase in concrete strength, failure after peak loading becomes more brittle, and the fracture energy in pull-out tests (which, as noted in [50], should be considered as a mixed mode) is significantly higher compared to pure Mode I.

Jonak et al., in study [56], showed that the fracture trajectory of rock under the influence of an undercut anchor depends significantly on the rock’s strength parameters, internal structure, and the geometric parameters of the anchor head. Research indicates that the free pull-out of a conical-headed undercut anchor is accompanied by a force component acting on the rock perpendicular to the anchor axis [56]. As a result, the fracturing process exhibits a multimodal nature. The axial force component leads to Mode I fractures (tensile rock failure), while the perpendicular component promotes the occurrence of Mode II fractures (shear-slip or sliding) [57].

Consequently, the fracturing process under the influence of undercut anchors is complex, resulting in diverse fracture trajectories ranging from paraboloidal to hyperboloidal shapes with curvature [56,58]. The ratio of the extent of detachment zone which rests on the free surface to the embedment depth (z/*h*_ef_, e.g., z_1_/*h*_ef1_, Figure 1) can reach values significantly greater than 1.5, as assumed in the CCD method [59,60].

In field testing conditions, a more convenient and practically useful approach for determining the angle α involves using the average extent of the detachment zone on the free surface of the medium [30]. However, the values obtained this way may differ from those determined using the CCD method.

Given the limited clarity in the available literature about the impact of undercut anchors having effective embedment depths on the extent and volume of detachment, conducting both experimental and numerical studies is essential to address these uncertainties.

In light of the aforementioned discrepancies, this study aimed to investigate the development of the failure cone zone as a function of the embedment depth and the diameter of the undercut anchor head. This objective was pursued through a numerical experiment using the FEM software ABAQUS (Abaqus 2023, Dassault Systems Simulia Corporation, Vélizy-Villacoublay, France). The findings from the numerical simulations were validated by comparing them with results obtained from a field experiment conducted in sandstone block quarries [30,61,62].

## 2. Materials and Methods

### Numerical Analysis

Numerical analysis was conducted on the interaction of a series of undercut anchors of the HDP-A type with the rock medium (gray sandstone) during classical anchor pull-out. The FEM analysis was performed using the ABAQUS software (Abaqus 2023, Dassault Systems Simulia Corporation, Vélizy-Villacoublay, France), employing the XFEM algorithm [63,64,65,66,67].

For the analysis of rock medium cracking due to the action of the breakout anchor, typical procedures, conditions of initiation, and the development of damages, using the FEM ABAQUS software, the following aspects were were used [68,69,70,71,72]:

- Damage initiation in rock material: Maximal Principal Stress;
- Damage evolution: type: energy, softening linear;
- Damage for traction—separation laws: Maximal Principal Stress Damage;
- Fracture Energy *E*_Ic_ = 0.355 N/mm;
- Damage stabilization—cohesive, stabilization coefficient: 1 × 10^−6^.

***Material properties of the rock medium that were adopted:***

Young’s modulus = 14,275 MPa,

Poisson’s ratio = 0.247.

Value of tensile failure stresses f_t_ = 7.74 MPa,

The analysis focused on the sandstone obtained from the quarries Braciszów and Brenna in Poland and the porphyry from the quarry Zalas in Poland. Rock samples were obtained during the “in situ” pull-out tests at the mentioned quarries, which were carried out as a part of the RODEST project financed by the Polish National Center of Science (RODEST No. 2015/19/B/ST10/02817) [73,74]. Each time, the rock material was collected from the testing site for the laboratory tests at the laboratories of the Department of Geomechanics and Underground Construction of the Faculty of Mining, Safety Engineering and Industrial Automation at the Silesian University of Technology. The parameters adopted for FEA analysis are within the range obtained from laboratory experiments [30,68]. In uniaxial compression and three-point bending tests [75], the Young’s modulus and Poisson’s ratio were determined for samples obtained from field studies. The experiments were conducted using an MTS 319.25 testing machine, with measurements of vertical and horizontal strains. Destructive testing was carried out on a Walter+Bai testing machine.

Destruction model (according to ABAQUS procedure)—Max Principal Stress: In order to simulate crack propagation under linear elastic conditions, the crack path direction must be determined. There are several methods used to predict the direction of crack trajectory such as the maximum tangential stress theory (or the maximum circumferential stress theory), the minimum strain energy density theory and the maximum energy release rate criterion based on Griffith’s theory.

***Anchor material:***

Steel: elastic; isotropic; elastic modulus—E = 210,000 MPa; Poisson’s ratio—ν = 0.3.

The friction coefficient of the steel head and the rock was assumed to be μ = 0.2.

**Figure 2:**
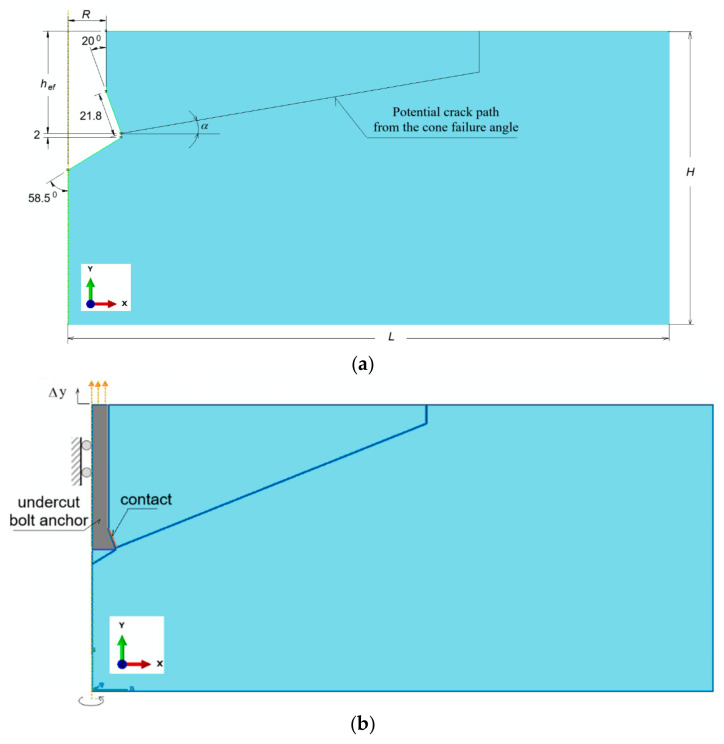
Axially symmetric model of rock medium: (**a**) dimensions of rock model with undercut for anchor; *R*—radius of cylindrical part of anchor; *h*_ef_—effective depth of anchorage; H, L—dimensions of rock medium model; α—angle of potential crack propagation (potential destruction cone); (**b**) “contact”—contact zone between rock and conical part of anchor head; Δy—displacement forcing of anchor.

A displacement-type loading was applied—displacement Δy was assigned to the anchor (Figure 2b) along the OY-axis of the adopted coordinate system, within the range Δy = 0 to 10 mm, with an assumed increment of displacement at each subsequent step of the simulation.

***Geometry of the rock medium model:***

The geometric model of the rock used in the study had dimensions of 800 mm × 400 mm. It was adopted as a rectangle with dimensions of *L* = 800 mm horizontally and *H* = 400 mm vertically (Figure 2a). A hole for the anchor was made at the axis of rotation, sized according to the considered anchor dimensions. As a result, models were analyzed based on characteristic dimensions, in the following forms: depth *h*_ef_ = 50, 100, 150, 200 mm.

The dimension *L* of the rock model is derived from the maximum extent of delaminations observed in field studies [30] on the free surface for the maximum anchoring depths *h*_ef_. Furthermore, adopting such a large dimension *H* in proportion to *h*_ef_ ensures that the supports of the pull-out-testing device do not influence the stress distribution generated by the anchor in the rock. The selected height of the sample *H*, in turn, guarantees that for the maximum anchoring depths considered in the FEM analysis, there will be no potential bending of the rock model beneath the anchor (as accounted for in computational models of undercut anchors, e.g., [1]).

For the assumed width of the rock model *L* and even the maximum planned anchoring depth, the *L*/*h*_ef_ ratio reaches a minimum value of 4. This is sufficient to avoid any potential disruptive influence of the anchor pull-out device’s supports on the stress distribution in the area of interest within the rock model (where the crack propagates).

The hole radius R for the anchor was set as follows: for M12 = 11 mm; M16 = 15 mm; M20 = 18.5 mm.

A flat axisymmetric model was used. For the anchors, a constant length of the generatrix of the undercut cone head was assumed, equal to 21.8 mm, with the cone angle of the head set at 20°.

In the zone of interactions of anchor elements and the rock (Figure 2b), the “surface-to-surface” procedure, and contact with friction of type “Penalty contact”, available in the ABAQUS software, were used. In all cases, a constant length was assumed for the form of the conical undercutting head (=21.8 mm, as in Figure 2a). As a result, the length of the contact zone was also constant in all cases analyzed.

***Boundary conditions***

**Figure 3:**
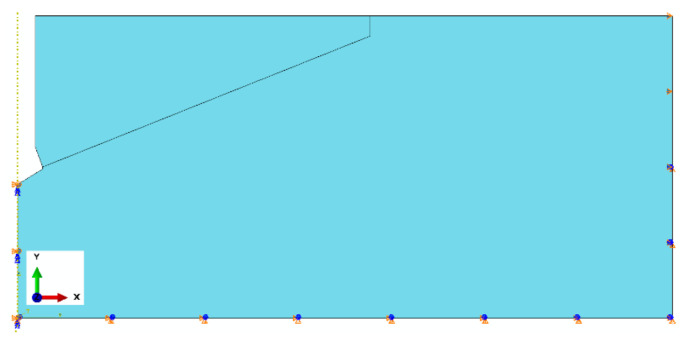
Restraints of the boundary nodes of the rock medium model.

For the rock medium model, restraints were assumed:

Left edge (axis of symmetry of axisymmetric model): U1 = U3 = UR2 = 0;

Bottom edge U1 = U2 = UR3 = 0;

Right edge in the part below the anchoring depth—U1 = U2 = UR3 = 0, upper part of U1 = 0. The method of restraining the edges of the rock model is illustrated in Figure 3.

***Sensitivity Analysis of the Rock Model to Finite Element Mesh Size***

To determine the influence of finite element sizes on the model’s response, a sensitivity analysis was performed. The study presents sample results of this analysis for an M20 anchor with an effective embedding depth of *h*_ef_ = 150 mm.

**Figure 4:**
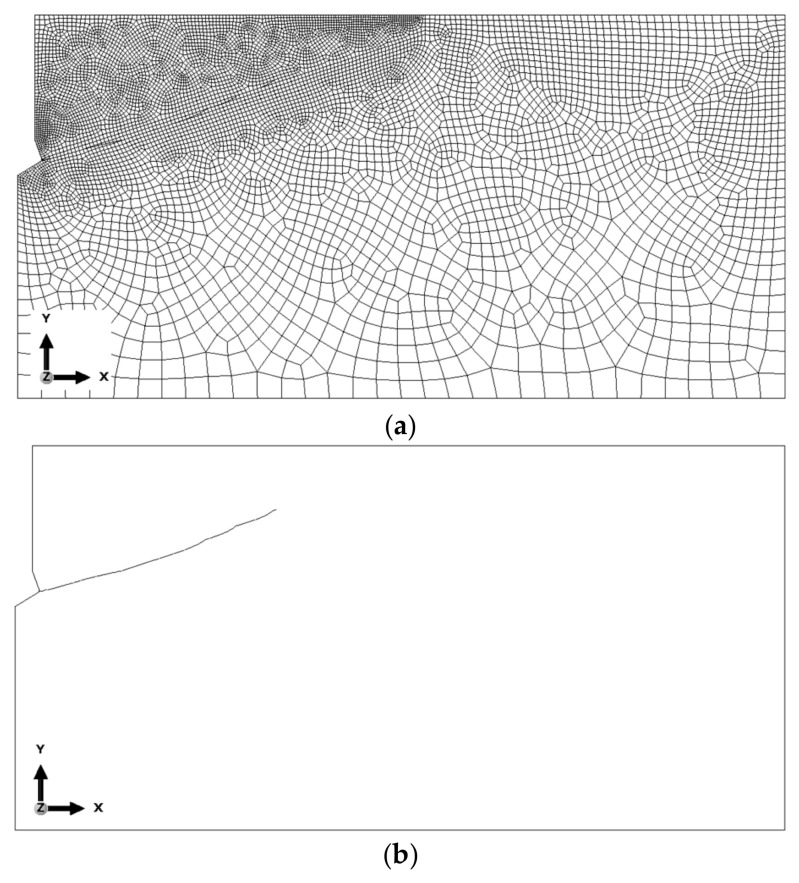

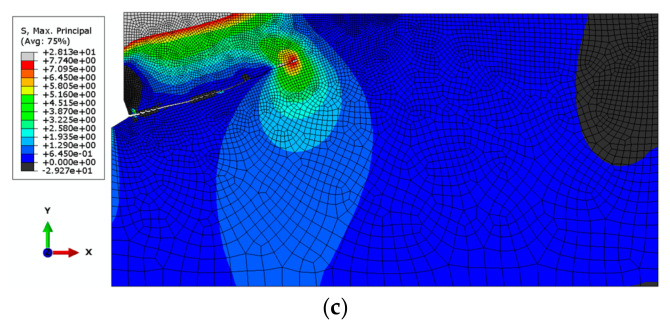
Results of the analysis conducted for the base mesh: (**a**) base mesh model, (**b**) obtained crack propagation trajectory, and (**c**) distribution of maximum principal stresses *σ*_max_ near the anchor head and crack tip.

The global element size of the mesh was set as follows:

(a) Base mesh—the side length of the elements was set to 25 mm. In the contact area with the anchor, element sizes ranging from 2 to 5 mm were analyzed. Along the upper edge of the model, the element size varied from 2 to 10 mm. Along the predicted crack path, the base length of the elements was set to 4 mm. The mesh was composed of Axisymmetric Stress, linear, reduced integration, and 4-node type (CAX4R) elements. As a result, the total number of nodes was 8024, and the total number of elements was 7797 (for the M20 anchor, *h*_ef_ = 150 mm). Figure 4 illustrates the results obtained (finite element mesh, crack propagation trajectory, and the plot of maximum principal stress distribution *σ*_max_.

(b) Mesh with Reduced Element Size

**Figure 5:**
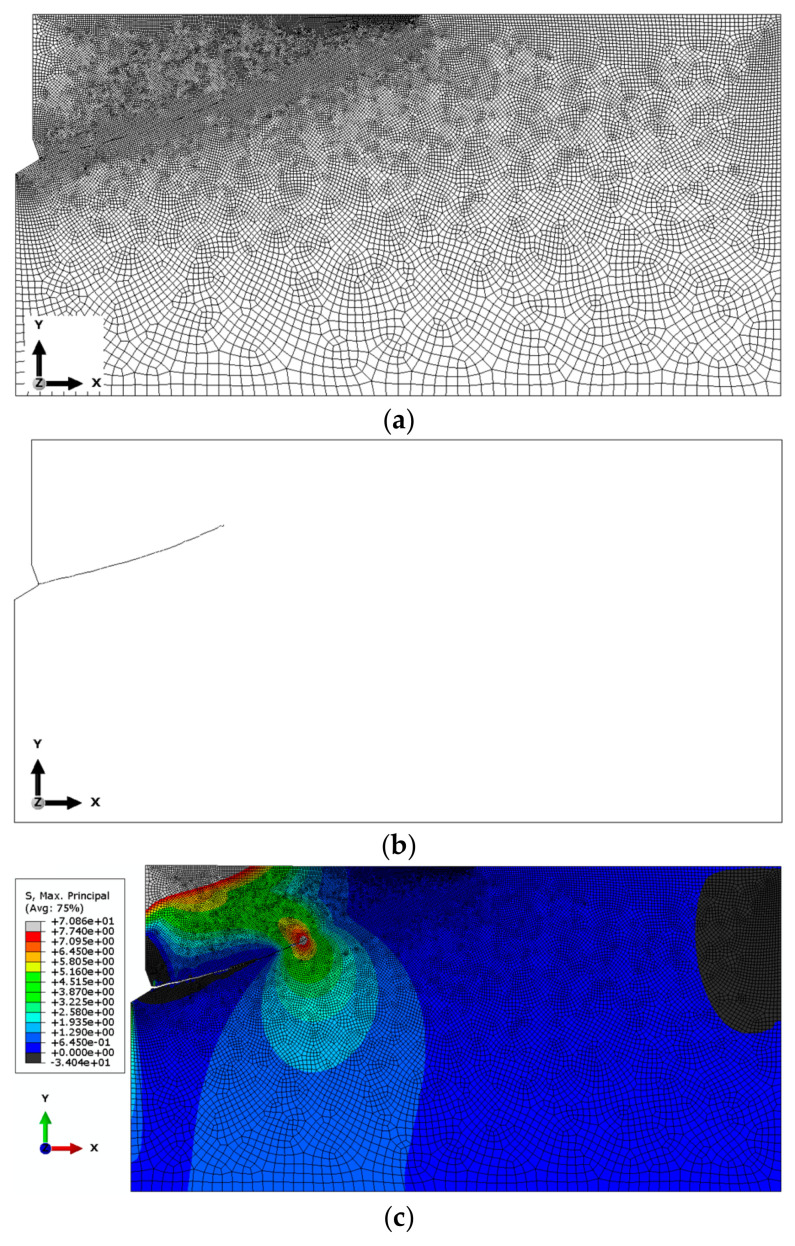
Results of the analysis conducted for the mesh with reduced element dimensions (base element size × 0.5): (**a**) base mesh model, (**b**) obtained crack propagation trajectory, and (**c**) distribution of maximum principal stresses *σ*_max_ near the anchor head and crack tip, using the mesh with reduced element dimensions.

Figure 5 presents the results obtained for the mesh with reduced element dimensions (scaled to 0.5 of the base dimensions). The global element size was 12.5 mm, with element sizes in the anchor contact area ranging from 1 to 2.5 mm. Along the edges in the upper part of the structure, the element size varied from 1 to 5 mm, while along the predicted crack path, the base length of the elements was 2 mm. The mesh was composed of Axisymmetric Stress, linear, reduced integration, 4-node type (CAX4R) elements. The total number of nodes was 30,767, and the total number of elements was 30,403 (for the M20 anchor, *h*_ef_ = 150 mm).

(c) Mesh with enlarged element dimensions

**Figure 6:**
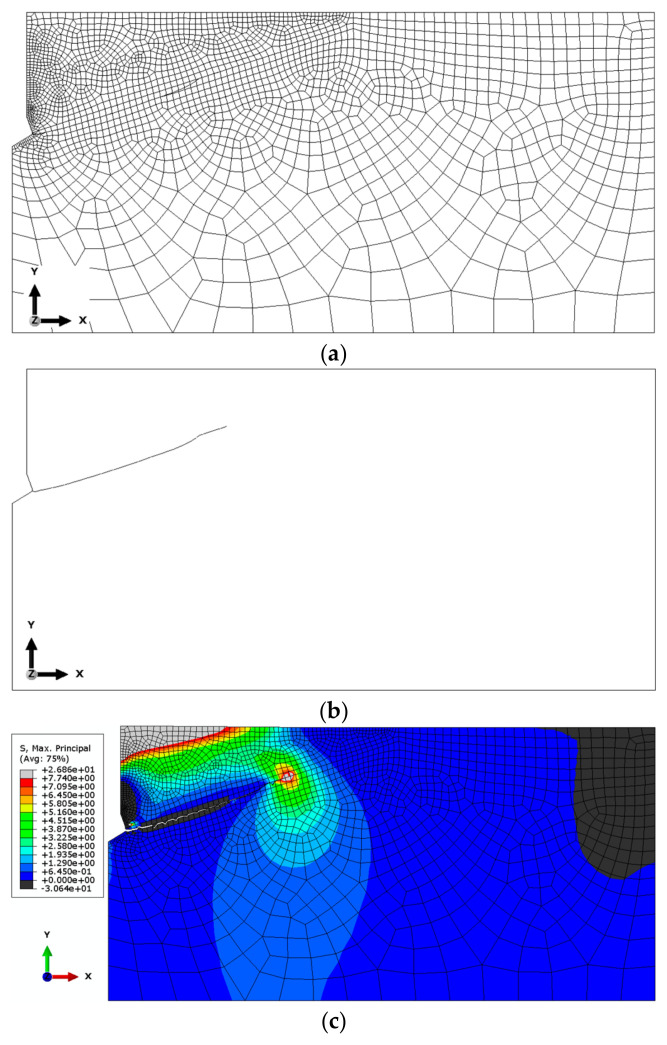
Results of the analysis conducted for the mesh with enlarged element dimensions (base element size × 2): (**a**) base mesh model, (**b**) obtained crack propagation trajectory, and (**c**) distribution of maximum principal stresses *σ*_max_ near the anchor head and crack tip.

Figure 6 presents the results obtained for the mesh with base element dimensions scaled by a factor of 2.

Based on the results of the sensitivity analysis, the most suitable mesh was determined to be the one proposed as the “base mesh” (Figure 4a). This mesh satisfactorily represents the crack propagation trajectory (a smooth trajectory without “jagged” steps, as shown in Figure 6c) while significantly reducing the computational time required for the algorithm to complete.

***Finite element mesh***

**Figure 7:**
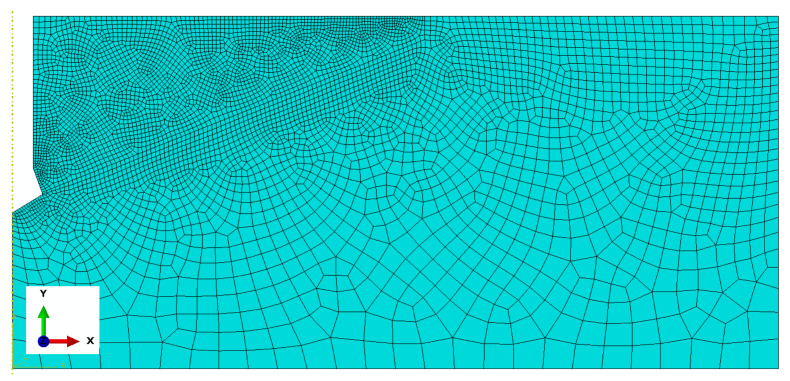
Finite element mesh of the rock medium model.

The model of the rock medium was described by a finite element mesh as in Figure 7. This mesh was finally adopted, as a result of a previously conducted sensitivity analysis of the model to finite element size and previous studies [56,76,77,78,79]. In view of the calculation time and smoothness of the fracture trajectory, a variant was adopted where the global mesh size was 25 mm; in the anchor contact area, it was 2 to 5 mm, for the edges in the upper part of the solid the mesh size was assumed variable from 2 to 10 mm, and along the line of the predicted fracture 4 mm was assumed. Element meshes of the following types (according to ABAQUS nomenclature) were used: Axisymmetric Stress, linear, reduced integration, 4-node CAX4R. The number of nodes ranged from 4680 (M12 anchor, *h*_ef_ = 50 mm) to 8683 (M20 anchor, *h*_ef_ = 200 mm). The number of finite elements then ranged from 4538 to 8507, respectively.

## 3. Results and Discussion

The authors of this study are engaged in numerical simulations of brittle rock failure during the mechanical destruction of rock medium structures [80]. As part of their research, they have proposed the use of undercut anchor pull-out processes for detaching rock blocks [76,77,81]. This process is considered an alternative to blasting methods or for use in specific mining conditions, such as rescue operations in confined spaces or environments with methane or coal dust. Due to the regulatory restrictions and standards imposed by the EU, this issue has garnered growing interest among mining engineers and civil engineering specialists.

Understanding the mechanism of rock medium failure during the installation and pull-out of the studied anchors can also contribute valuable knowledge to other technical fields, such as civil engineering, given the widespread use of undercut anchors. To eliminate the need for a massive support structure to secure the pulling actuator used to detach rock elements [52,61,62], an alternative method of generating destructive stresses in the rock medium was proposed. This method involves expanding the undercut anchor head using a drive screw supported at the bottom of a borehole drilled into the rock [57,78].

A key factor in popularizing the proposed detachment technology is determining the influence of effective embedment depth (*h*_ef_) on the development of fracture trajectories (parameters of the potential rock failure cone), which directly relate to the volume of rock blocks detached by the anchor. This volume enables the calculation of process efficiency indicators and the selection of optimal parameters for the detachment process, considering both the detachment force (or torque applied to the anchor) and the volume of detached rock blocks [82].

### 3.1. Numerical Analysis

**Figure 8:**
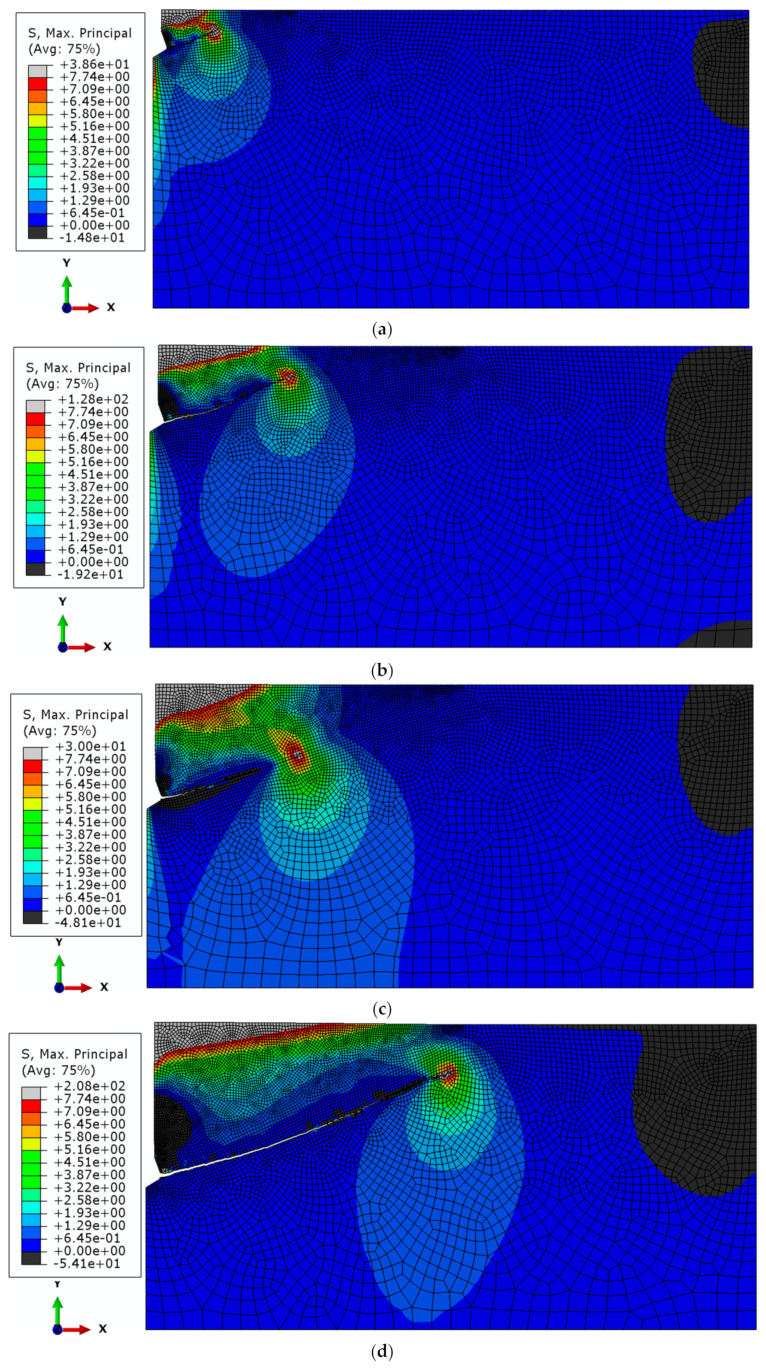
Fracture trajectory (failure cone) and maximum principal stress distributions *σ*_max_ for the M12 anchor, for: *h*_ef_: (**a**) 50, (**b**) 100, (**c**) 150, (**d**) 200 mm.

The fracture trajectory of the rock medium model under the influence of the M12 anchor, along with the local stress distribution, for various anchoring depths, is shown in Figure 8.

As described earlier in the material data, the maximum principal stress (Rankine’s criterion) was selected as the parameter responsible for material failure. The following material parameters were applied: tensile strength, *f*_t_ = 7.74 MPa, and critical strain energy release rate, G_Ic_ = 0.355 N/mm. The tensile strength *f*_t_ also represents the stress that initiates crack propagation at the crack tip.

**Figure 9:**
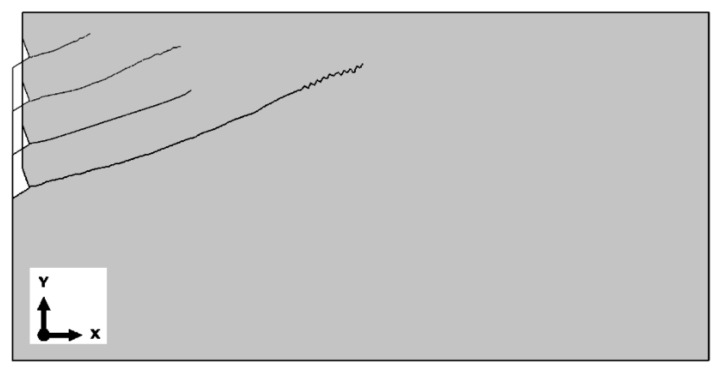
Collective summary of fracture trajectories for the M12 anchor: *h*_ef_ = 50, 100, 150, 200 mm.

To facilitate comparisons, Figure 9 presents a collective summary of the fracture trajectories obtained for the M12 anchor at the considered anchoring depths. The presented trajectories were recorded up to the point at which the ABAQUS program terminated the calculations, either due to exceeding the allocated computation time or, more commonly, due to the inability to achieve algorithm convergence.

**Figure 10:**
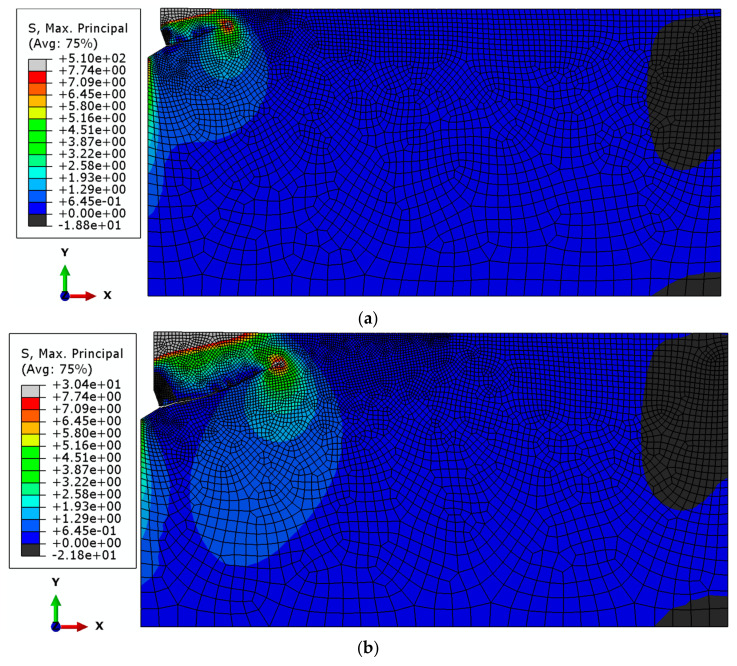

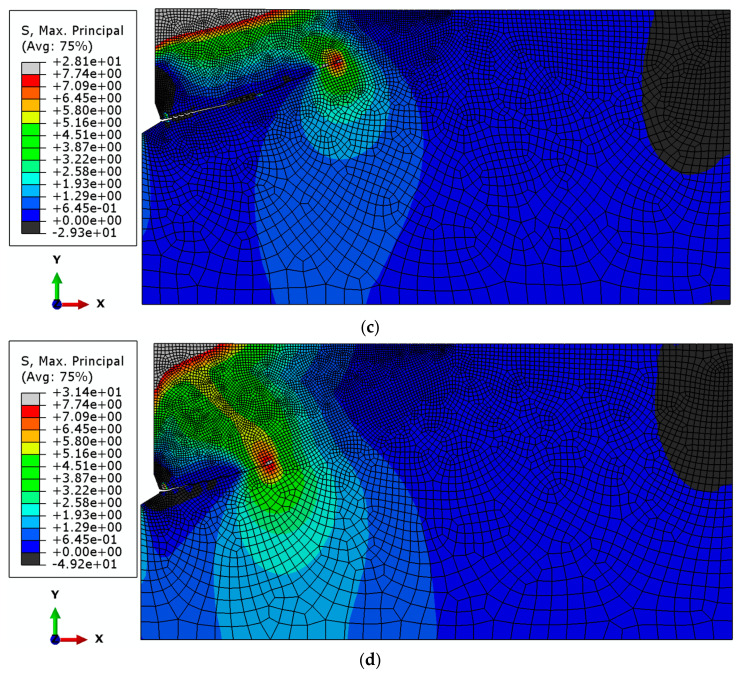
Fracture trajectory (failure cone) and maximum principal stress distributions *σ*_max_ for the M20 anchor, for *h*_ef_: (**a**) 50, (**b**) 100, (**c**) 150, (**d**) 200 mm.

In turn, Figure 10 illustrates the formation of the failure cone (fracture trajectory) for the M20 anchor for comparison purposes.

The maximum tensile stress occurs at the crack tip (red color) and corresponds to the tensile strength of the rock medium, f_t_ = 7.74 MPa. The gray color (at the crack tip) indicates a higher value resulting from the approximation of tensile stresses *σ*_max_ exceeding this value, which in turn stems from the tolerance settings in the ABAQUS software and the automatic discretization of the obtained stress range into bands visible on the stress maps. The black band of compressive stresses observed in the lower right corner may result from the local distribution of geometric parameters describing the geometry of the rock model and the anchor at a given anchoring depth. Similar effects have been observed in other studies [52].

**Figure 11:**
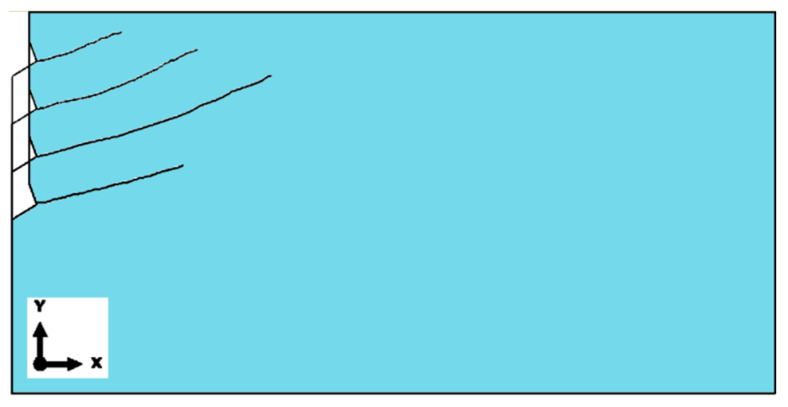
Influence of effective anchoring depth, *h*_ef_ = 50, 100, 150, and 200 mm, on the formation of the failure cone for the M20 anchor.

Similarly to the earlier analyses, to facilitate comparisons, Figure 11 presents a collective summary of the fracture trajectories of the rock medium under the influence of the M20 anchor.

**Figure 12:**
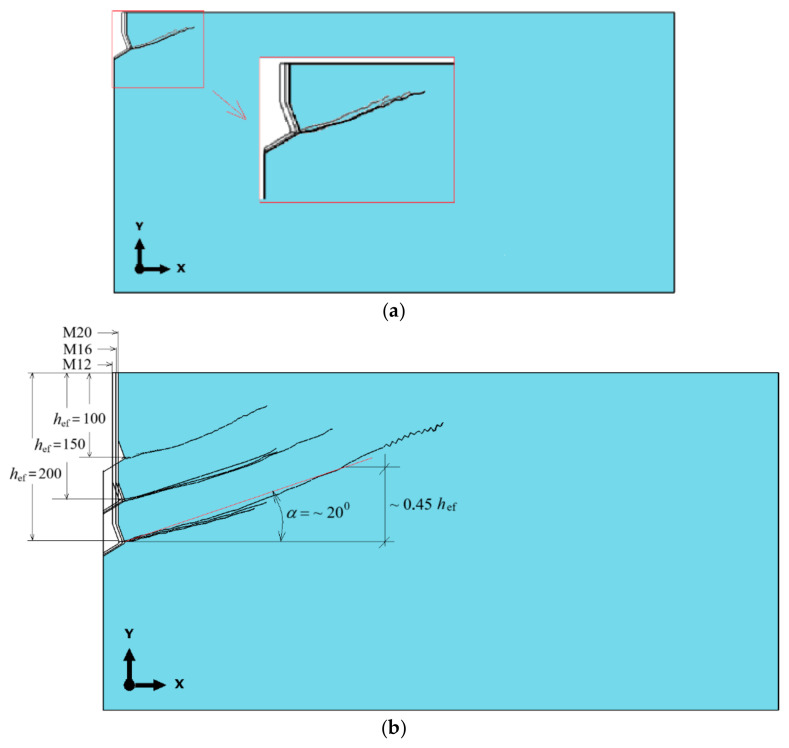
Fracture trajectories for M12, M16, and M20 anchors for the following conditions: (**a**) *h*_ef_ = 50 mm, (**b**) *h*_ef_ = 100, 150 and 200 mm.

To provide a more comprehensive illustration of the influence of anchoring depth and the diameter of the undercut anchor head, a summary of the obtained analysis results has been compiled (Figure 12).

The obtained fracture trajectory patterns indicate that, for the adopted material model and fracture criterion of the rock medium (available in the ABAQUS software), the anchoring depth of undercut anchors does not have a significant effect on the trajectory or extent of the fracture propagation (and, consequently, the potential failure cone). Similarly, no significant influence of the undercut anchor head diameter on the fracture trajectory was observed, regardless of the anchoring depth, as shown in Figure 12.

The fracture trajectories, within the range where the computational algorithm achieves convergence, exhibit similar patterns, particularly during the initial phase of rock medium failure development. From the perspective of the research team’s interest in the formation of the detached volume of rock during undercut anchor pull-out, the only critical parameter is the effective anchoring depth *h*_ef_.

This finding corroborates relationships established in studies such as [52], which also demonstrated no significant influence of the undercut anchor diameter on the potential extent of the failure zone. As shown in Figure 12b, the average failure cone angle α (determined in a manner similar to the CCD method recommendations) was approximately 20°.

### 3.2. Experimental Validation

Pull-out tests for undercut anchors conducted under field conditions in quarries extracting rock aggregates are described in detail, for example, in [30,61]. After each anchor pull-out test, the surface failure trace remaining in the rock medium was scanned using an optical scanner. Subsequently, through digital processing of the point cloud measurements, the shape and geometric parameters of the failure surface (pseudo-cone of failure) were reconstructed [61]. The tests utilized standard undercut anchors of the HILTI HDA-P type and dedicated pull-out testing equipment (designed by ITG KOMAG [61]). Experiments were carried out in sandstone and porphyry. As a result of the tests carried out within the project, 115 successful solid rock loosening trials were performed using an undercut anchor. The anchoring depth *h*_ef_ was in the range of 58–162 mm [30,73]. It should be borne in mind that the implementation of experiments in the conditions prevailing in mines is very difficult (it is difficult to ensure the constancy of the parameters of the rock mass. In a rock mass with comparable physical and mechanical conditions, it was possible to realize 6–8 repetitions of a given type of anchor pullout test.

The failure surface was scanned using a 3D digital laser scanner. The point cloud obtained from the scan was manually processed and converted into an STL triangulated surface. Subsequently, specialized LEIOS 2 R10 software (E.G.S. Srl, 2019, Bologna, Italy) was employed to further process and convert the STL model into a *.sat model. The 3D solid model of the failure surface after the breakout failure was then processed using Inventor software to derive a breakout cone element. Data from the 3D solid model of the breakout cones were used to calculate the failure surface areas, the cone surface areas, and the volume of the breakout cone [59].

**Figure 13:**
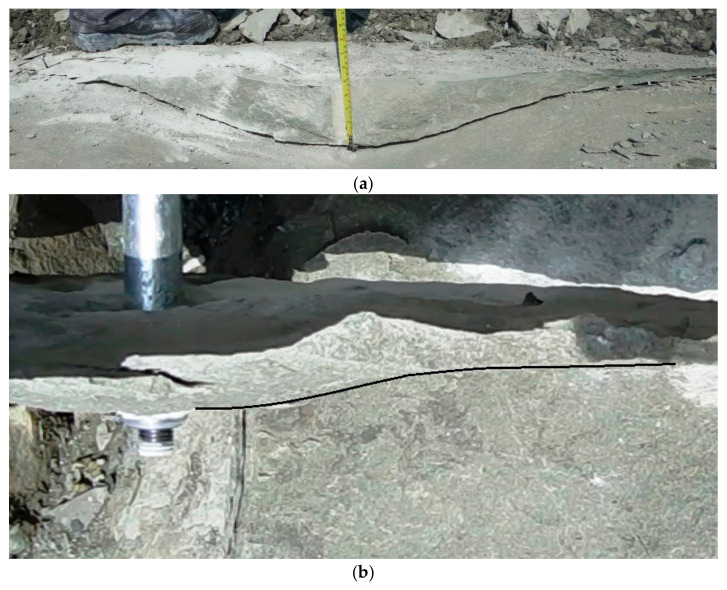

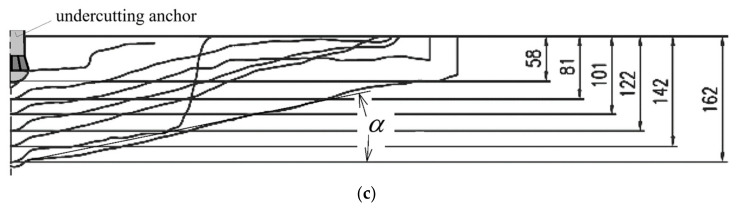
Real propagation of the loosening path, sandstone, in the light of own tests—(**a**,**b**); the crack propagation for a single-undercutting anchor, depending on the embedment depth—Brenna Mine—(**c**).

In axial cross-sections of the failure cone taken through the axis of the anchor hole, fracture trajectories were obtained [73,83], as illustrated in Figure 13 for gray sandstone (Brenna Mine).

Figure 10 [73] indicates that within the range of stable crack propagation (the destruction zone), the obtained fracture trajectories in gray sandstone are nearly identical, regardless of the effective anchoring depth *h*_ef_ or the diameter of the anchor head. The average value of the cone failure angle α ranged between 13° and 17° [62].

The results of field studies allowed for the positive validation of numerical analysis results conducted using the Finite Element Method (FEM). For the adopted material assumptions of the rock medium, geometric parameters of the undercut anchor, and within the range of analyzed anchoring depths, it was demonstrated that neither an effective anchoring depth nor the anchor head diameter influenced the trajectory of fracture propagation in the rock medium (gray sandstone). The obtained trajectories were very similar in shape to those derived from FEM analysis.

The actual cone failure angles α (13–17°) were slightly smaller than those obtained in FEM numerical analysis (~20°). These differences may stem from the assumed friction coefficient μ between the rock and the anchor head, the precise value of which is challenging to determine for specific field testing conditions due to the numerous influencing factors (e.g., rock moisture, etc.).

### 3.3. Limitations and Future Plans

The presented results of analyses and experimental studies primarily pertain to:

- the interaction of undercut anchors with the rock medium,
- rocks treated as a homogeneous, isotropic-medium and devoid of heterogeneities and fractures.

In typical rock media, the destruction mechanism of the structure is more complex than depicted in this study, leading to differences in the description of failure forms and the relationships governing, for example, the pull-out force of the anchor [84,85,86].

Due to the significant differences observed in the load transfer mechanism of undercut anchors compared to “headed bolt” anchors (e.g., [87]), the obtained results cannot be generalized or directly compared to other anchor designs. This limitation applies to certain aspects of methods such as CCD.

In numerous studies concerning “headed bolt” anchors (e.g., [48]), a critical comparative parameter for evaluating anchor interaction with the medium is anchor stiffness, often defined by the ratio of the anchor head diameter to the effective anchoring depth, or the ratio of the anchor head diameter to the diameter of its shank/rod. In the analysis presented here, an important assumption was the assumption of constant lengths of the undercutting head cone (as well as the contact zone of the head with the rock). Such an analytical approach can result in different outcomes.

The authors plan to continue and expand the study to include and analyze both in more homogeneous media, such as concrete of a certain size or aggregate distribution, and for other rock types. Field and numerical studies using other new anchor types are also planned. We have also planned to conduct analyses for multiple anchor systems and to perform analyses in spatial systems.

## 4. Conclusions

Numerical research results, positively validated through field studies, demonstrated that within the examined parameter range for undercut anchors, neither the effective anchoring depth *h*_ef_ nor the diameter of the anchor head significantly influence the angle of the failure cone. Consequently, the potential volume of detached rock primarily depends on the effective anchoring depth *h*_ef_.

The results of numerical analyses are highly consistent with those obtained during field studies, as clearly illustrated in Figure 10. The results also align with other numerical studies conducted by members of the research team, particularly [69]. The limitations of the XFEM algorithm in ABAQUS, which prevent tracking the final phase of failure propagation, as described in [88,89], make it impossible to develop definitive, digital comparative indicators in this regard.

The presented results significantly expand the current understanding of the failure mechanisms of media subjected to undercut anchors. Previous reports primarily focus on media such as concrete, which does not always accurately describe the behavior of rock materials. The findings contribute new insights and verify existing reports on the failure mechanisms of brittle media, particularly regarding the influence of anchoring depth on the extent of potential rock damage (i.e., the geometry of the so-called failure cone). This aspect has generally been overlooked in the literature, and the available information in this area has been unreliable.

Due to the limitations of the current research, further studies are required to fully understand the rock detachment process. These studies should aim to determine the impact of additional physical factors (including anchor stiffness) and the heterogeneity of the rock medium structure on the progression and extent of detachment under the influence of undercut anchors.

## Data Availability

The data presented in this study are available on request from the corresponding author due to project implementation constraints.
